# CD44v6 expression in non-anaplastic thyroid carcinoma: characterization of candidates for targeted therapy

**DOI:** 10.1186/s13044-025-00266-3

**Published:** 2025-10-03

**Authors:** Adam Stenman, Joachim N. Nilsson, Vincenzo Condello, Anja C. L. Mortensen, Marika Nestor, Jan Zedenius, C. Christofer Juhlin

**Affiliations:** 1 Department of Molecular Medicine and Surgery, Karolinska Institutet, Stockholm, Sweden; 2 Department of Breast, Endocrine Tumors and Sarcoma, Karolinska University Hospital, Stockholm, Sweden; 3 Department of Nuclear Medicine and Medical Radiation Physics, Karolinska University Hospital, Stockholm, Sweden; 4 Department of Oncology-Pathology, Karolinska Institutet, 171 76 Stockholm, Sweden; 5 Department of Immunology, Genetics and Pathology, SciLifeLab, Uppsala University, Uppsala, Sweden; 6 Department of Pathology and Cancer Diagnostics, Karolinska University Hospital, Stockholm, Sweden

**Keywords:** CD44v6, Immunohistochemistry, Thyroid cancer, Theranostics, Therapy

## Abstract

**Background:**

CD44v6 is a membranous antigen upregulated in solid tumors and a promising molecular radiotherapy target, especially in anaplastic thyroid carcinoma (ATC). A Phase 1 trial recently launched to evaluate the lutetium-labeled anti-CD44v6 antibody [^1^⁷⁷Lu]Lu-DOTA-AKIR001 in CD44v6-positive solid tumors. Given limited data in non-ATC, we assessed CD44v6 immunoreactivity in tumors that may progress to a radioiodine-refractory state.

**Materials and Methods:**

An exploratory cohort of 33 tumors (30 papillary thyroid carcinomas [PTCs], 3 poorly differentiated thyroid carcinomas [PDTCs]) was screened using the VFF-7 antibody, supported by detailed iodine concentration, genetic, and RNA sequencing data. A validation cohort of 40 oncocytic thyroid carcinomas (OTCs), 28 additional PDTCs, and one differentiated high-grade thyroid carcinoma was also screened using two antibody clones, VFF-7 and VFF-18.

**Results:**

In the exploratory cohort, 10 of 33 tumors (30%) showed focal or diffuse CD44v6 expression, while the rest were negative. Among OTCs in the validation cohort, 15 of 40 (38%) were partially or diffusely positive, and in PDTCs, 14 of 28 (50%) showed focal or diffuse staining. The VFF-7 and VFF-18 clones produced similar patterns.

**Conclusions:**

Substantial subsets of non-ATCs express CD44v6, indicating that some patients may be candidates for [^1^⁷⁷Lu]Lu-DOTA-AKIR001 radiotherapy, particularly when conventional treatments are exhausted.

**Supplementary Information:**

The online version contains supplementary material available at 10.1186/s13044-025-00266-3.

## Introduction

CD44v6 is an isoform of the CD44 cell surface glycoprotein generated through alternative splicing, incorporating variant exon 6 into the extracellular domain. This variant has been implicated in key tumor-related processes, including cell adhesion, migration, invasion, and resistance to apoptosis [1]. By interacting with growth factor receptors and components of the extracellular matrix, CD44v6 can activate signaling pathways that contribute to tumor progression and metastasis. Its limited expression in normal tissues, combined with upregulation in various malignancies, has made CD44v6 a promising biomarker and therapeutic target—particularly in tumors that are refractory to standard treatments [1–6]. Antibody-based approaches directed against CD44v6 offer a promising avenue, with applications both in molecular imaging and targeted therapy. Although early clinical attempts, such as bivatuzumab mertansine, were hampered by off-target toxicity, recent progress in antibody engineering and immuno-PET technology has renewed interest in this strategy. Building on these developments, new generations of fully human antibody fragments against CD44v6 have demonstrated encouraging tumor-targeting potential, though key translational questions—including immunogenicity and appropriate preclinical models remain to be addressed [7–9].

Previous mRNA and protein studies have demonstrated CD44v6 expression in a broad spectrum of thyroid cancers, ranging from well-differentiated follicular cell-derived carcinomas to aggressive subtypes such as poorly differentiated (PDTC) and anaplastic thyroid carcinoma (ATC) [10–16]. However, these studies have often lacked the integration of detailed clinical context, including treatment response, radioiodine avidity, and comprehensive molecular profiling.

In light of the recent initiation of a Phase I clinical trial investigating the safety and efficacy of the Lutetium-177 labeled anti-CD44v6 antibody [^1^⁷⁷Lu]Lu-DOTA-AKIR001 in patients with CD44v6-positive solid tumors (ClinicalTrials.gov ID NCT06639191), we aimed to provide a more clinically grounded assessment of CD44v6 expression in thyroid carcinoma. To this end, we analyzed two complementary cohorts: (A) an exploratory cohort with detailed clinical annotation, including radioiodine uptake profiles and mutational data, and (B) a validation cohort composed of PDTC and oncocytic thyroid carcinomas (OTC)—entities frequently associated with diminished radioiodine uptake and distant metastases [17–19].

Importantly, CD44v6 immunoreactivity was assessed using two distinct antibody clones, VFF-7 and VFF-18, allowing us to evaluate the consistency and sensitivity of detection across different immunohistochemical reagents. These clones belong to the well-characterized VFF series, which are among the most widely published CD44v6 antibodies in both experimental and clinical contexts. VFF-18, in particular, has been extensively validated and even adapted for therapeutic purposes, while VFF-7 has shown robust performance in diagnostic immunohistochemistry [9, 20–24]. Using both clones allowed us to cross-validate staining results and mitigate clone-specific variability, thereby enhancing the consistency and reproducibility of our findings. By correlating CD44v6 expression with clinical and molecular features, our goal was to better define its potential as a therapeutic biomarker in thyroid carcinoma, particularly in cases with limited treatment options.

## Materials and methods

### Patient Cohort and Sample Collection

**Fig. 1:**
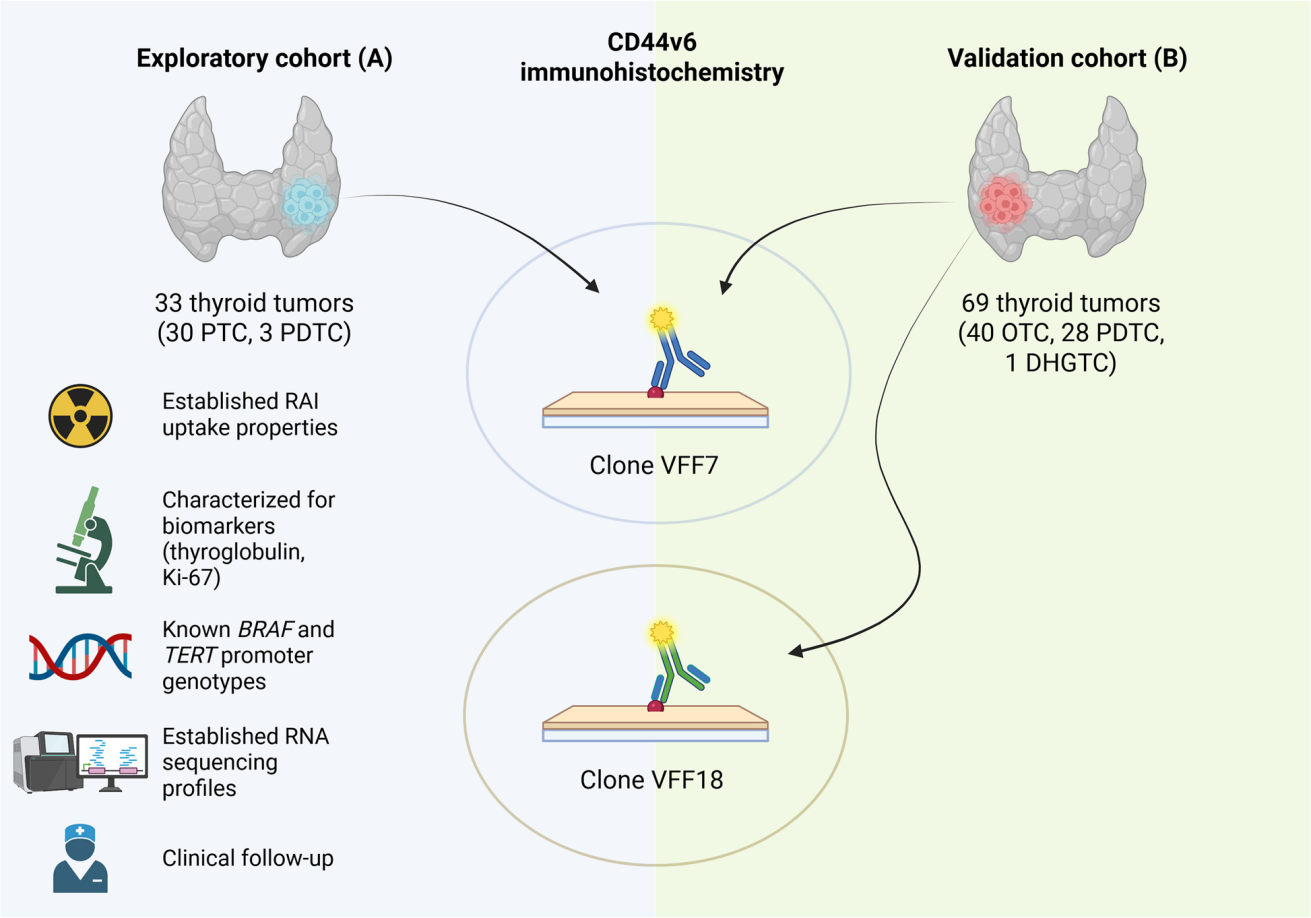
Overview of the study design. Created using BioRender.com

A schematic overview of the study design is presented in Fig. 1. A total of 102 non-ATC specimen were analyzed, including 30 papillary thyroid carcinomas (PTC), 31 PDTC, 40 OTC and one oncocytic differentiated high-grade thyroid carcinoma (DHGTC) collected from patients diagnosed at the Karolinska University Hospital between 2017 and 2021. All patients provided informed consent, and the study was approved by the Swedish Ethical Review Authority. The exploratory cohort (A) included 30 PTC and 3 PDTC tissue samples from 26 patients. From the seven patients contributing two samples, one sample from the primary tumor and one from a resected lymph node metastasis were analyzed. All cases in the exploratory cohort had detailed data on tumoral iodine concentrations based on preoperative administration of tracer doses, with subsequent ex vivo measurements following surgical resection [15, 25]. Additionally, these tumors had available somatic genetic data and RNA sequencing profiles, enabling correlation of CD44v6 expression with molecular and clinical parameters [26, 27]. The validation cohort (B) included 28 PDTC, 40 OTC (24 minimally invasive, 11 widely invasive, and 4 encapsulated angioinvasive OTCs), and one oncocytic DHGTC. All cases were re-reviewed by an endocrine pathologist to ensure that the nomenclature was consistent with the criteria laid out by the 2022 WHO classification of thyroid carcinoma [17].

### Immunohistochemistry

Immunohistochemical (IHC) staining for CD44v6 was performed on formalin-fixed, paraffin-embedded (FFPE) tissue sections using two different anti-CD44v6 antibody clones: VFF-7 (exploratory and validation cohort) (ab30436, clone VFF-7, Abcam, UK) and VFF-18 (validation cohort) (ab78960, clone VFF-18, Abcam, UK). We used two different clones to minimize clone-specific bias and to account for potential differences in epitope accessibility. While VFF-18 recognizes a centrally located epitope within v6 (aa 18–31), the precise residue-level epitope for VFF-7 has not been published. Using both reagents provided cross-validation of staining and mitigated conformational effects on single-clone detection.

FFPE sections (4 μm thick) were fixed to glass slides (Superfrost + , Thermo Scientific) and subjected to a 3-h incubation at 56 °C. After deparaffinization in xylene and rehydration in alcohol, heat-induced epitope retrieval was conducted using a Decloaking Chamber (Biocare Medical) at 110 °C for 5 min in Citrate buffer (pH 6, Sigma C-9999). To suppress endogenous peroxidase activity, a 30-min incubation with 0.15% hydrogen peroxide was carried out at room temperature, followed by a 30-min blocking step using 1% bovine serum albumin (BSA). Following optimization of primary antibody dilution, slides were incubated with the primary antibody overnight at + 4 °C in a humid chamber. The secondary biotinylated antibody was incubated for 30 min at room temperature. Subsequently, sections were exposed to avidin–biotin enzyme complex (Vectastain ELITE ABC kit HRP, PK-6100, Vector Laboratories) for 30 min. Visualization was achieved using a peroxidase substrate DAB kit (VectorI DAB SK-4100, Vector Laboratories) for 3 min. The sections were counterstained with Mayer's hematoxylin for 1 min, followed by dehydration through graded alcohols, xylene treatment, and cover slipping with Pertex (Histolab). The staining intensity and distribution were evaluated by an endocrine pathologist (CCJ) and categorized as positive, negative, or focal areas with immunostaining. Positive controls consisted of a de-identified case of mammary ductal adenocarcinoma, while negative controls were thyroid tissue with omission of the primary antibody. Breast carcinoma was selected as a comparative control because it represents a non-thyroid tissue that is well documented to consistently exhibit CD44v6 expression [28].

### Tumor characteristics and clinical outcomes

Tumor characteristics, including size, subtype, and presence of extrathyroidal extension, were recorded for each case. Clinical outcomes, including recurrence, metastasis, and death, were recorded for cohort (A) during a follow-up period that ranged from 0 to 4 years. The association between CD44v6 expression and clinical outcomes was analyzed.

### Radioiodine avidity

All tumor tissue in cohort (A) had iodine concentrations measured through ex vivo analysis after surgical resection. Representative tumor and healthy thyroid samples were subjected to radioactivity measurements following an injection of 5–10 MBq of ^131^I two days prior to surgery. Iodine concentrations were normalized to proportions of injected activity (IA) per gram tissue. Normalized iodine concentrations at two days post-injection have been shown to clearly correlate with the iodine avidity in subsequent metastases [15].

### RNA sequencing

Whole-transcriptome (RNA-seq) data from our previous study were analyzed to assess potential differences in gene expression patterns between CD44v6-positive and negative cases [27]. Differential expression analysis was performed using the DESeq2 R package [29], as previously described [27]. To further explore the relationship between CD44v6 status and global transcriptomic profiles, principal component analysis (PCA) was conducted. Moreover, the expression level of the standard variant of *CD44 (CD44s),* reported as transcript per million (TPM) in CD44v6-positive vs. negative cases was also analyzed.

### Statistical analysis

Statistical analysis was performed using SPSS software (version 25.0) and R (version 4.2.2, R-project.org). Welch’s t-test was used to compare iodine avidity and CD44v6 expression. Chi-squared test was used to assess the association between CD44v6 expression and clinicopathological variables. Cohen’s weighted kappa was used to assess correlation between the two antibodies used for CD44v6 staining. A p-value of < 0.05 was considered statistically significant.

## Results

### Exploratory cohort (A)

**Fig. 2:**
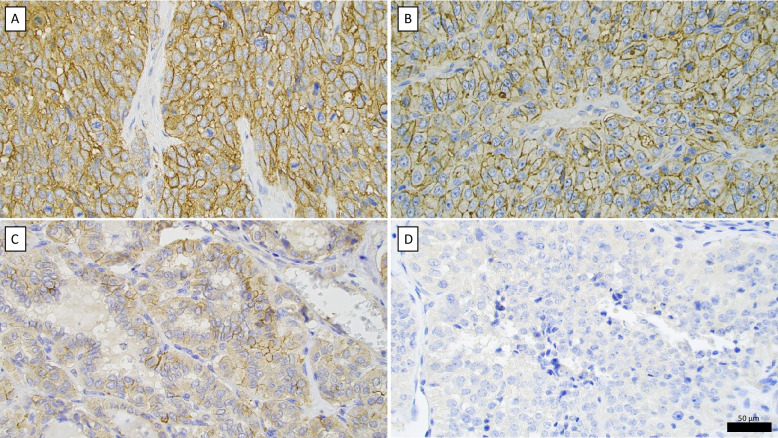
CD44v6 immunohistochemistry (clone VFF7). **A** De-identified mammary ductal adenocarcinoma specimen displaying strong membranous CD44v6 staining, fulfilling the criterion for “positive” staining. **B** Poorly differentiated thyroid carcinoma (PDTC) from the validation cohort with positive CD44v6 immunostaining. **C** Diffuse sclerosing subtype of papillary thyroid carcinoma from the exploratory cohort showing focal immunoreactivity, with tumor cells displaying both positive and negative membranous staining. **D** PDTC from the exploratory cohort with absent CD44v6 expression (termed “negative”). Scale bar in black is 50 µm

This cohort included 30 PTC and 3 PDTC with tumor sizes ranging from 10 to 75 mm, with a median size of 30 mm. In the PTC group, 10 out of 33 tumors (30%) demonstrated CD44v6 expression using the VFF-7 clone, either focal or diffuse (Table 1). Among the PDTC cases, 2 out of 3 tumors (67%) showed CD44v6 positivity. In all seven patients contributing both primary tumors and lymph node metastases to the analysis, CD44v6 staining results were concordant between the two sites (Fig. 2).

**Table 1:** CD44v6 immunohistochemistry and clinical characteristics of the patients included in the study

	**Exploratory Cohort (A)**	**Validation Cohort (B)**
***Parameter****s*		
**Sex**		
Male: Female (%)	10 (38%): 16 (62%)	44 (64%): 25 (36%)
**Age at diagnosis**		
Median (range) years	53 (19–81)	63 (16–86)
Mean (SD) years	54 (17)	61 (16)
**Tumor type**		
*Total, n (%)*	**33 (100%)**	**69 (100%)**
Papillary thyroid carcinoma, n (%)	30 (91%)	0 (0%)
Poorly differentiated thyroid carcinoma, n (%)	3 (9%)	28 (41%)
Oncocytic thyroid carcinoma, n (%)	0 (0%)	40 (58%)
Differentiated high-grade thyroid carcinoma, n (%)	0 (0%)	1 (1%)
**CD44v6 IHC**		
*VFF-7 Clone, n (%)*	33 (100%)	69 (100%)
- Positive, n (%)	1 (3%)	8 (12%)
- Negative, n (%)	23 (70%)	45 (65%)
- Focal, n (%)	9 (27%)	16 (23%)
*VFF-18 Clone, n (%)*	NA	69 (100%)
- Positive, n (%)	NA	17 (25%)
- Negative, n (%)	NA	39 (56%)
- Focal, n (%)	NA	13 (19%)

The iodine concentrations within the cohort ranged a thousand-fold, from the lowest detectable value of 2 × 10^–6^ IA/g to 2 × 10^–3^ IA/g. For reference, healthy thyroid tissue in the cohort had a median value of 2 × ­10^−2^ IA/g. There was no statistically significant difference between iodine avidity and CD44v6 expression (4.5-fold higher iodine avidity in tumors expressing any CD44v6, CI 0.78–24). Among cases with no observable iodine avidity, there were both CD44v6 positive and negative samples. However, there was a significant negative correlation between *BRAF* genotype and CD44v6 expression, meaning *BRAF*-mutated tumors were less likely to express CD44v6 (Chi^2^: 4.24, *p*-value: 0.039). Patients with CD44v6 positive tumors were significantly younger than those without expression (14 years younger, CI 2–24 years). No significant association between CD44v6 expression and risk of recurrent disease was observed (Chi^2^: 1.46, *p*-value: 0.23).

### Validation Cohort (B)

**Fig. 3:**
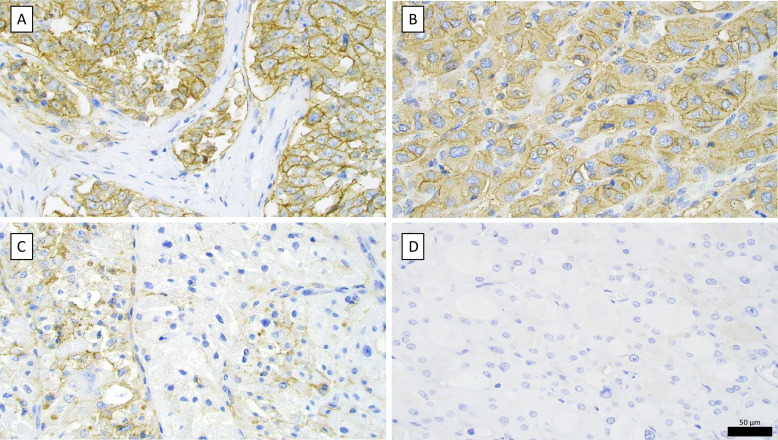
CD44v6 immunohistochemistry (clone VFF18). **A** De-identified mammary ductal adenocarcinoma specimen displaying strong membranous CD44v6 staining (“positive”). **B** Oncocytic thyroid carcinoma (OTC) of the validation cohort with positive CD44v6 immunoreactivity. **C** Poorly differentiated thyroid carcinoma of the validation cohort displaying focal CD44v6 positivity, exemplified here by subsets of tumor cells with clear-cut membranous signal. **D** OTC of the validation cohort with absent CD44v6 immunoreactivity. Scale bar in black is 50 µm

This cohort included at total of 69 high-grade non-ATC cases (28 PDTC, 40 OTC and one oncocytic DHGTC) with tumor sizes ranging from 9 to 120 mm, and a median size of 35 mm. Immunohistochemical staining for CD44v6 was performed using two antibody clones, VFF-7 and VFF-18. Among the PDTC cases, 14 out of 28 tumors (50%) showed either focal or diffuse positivity for CD44v6 (Table 1). In the OTC group, 15 out of 40 tumors (38%) were partially or diffusely positive for CD44v6 (Fig. 3). The sole DHGTC case was focally positive for VFF-7 and diffusely positive for VFF-18. The staining intensity and distribution were evaluated by the same expert pathologist (CCJ) as described in detail above and categorized as positive, negative, or focal areas with immunostaining.

The association between CD44v6 expression and clinical outcomes was analyzed, showing no significant impact of CD44v6 positivity on overall survival or disease-free survival.

**Fig. 4:**
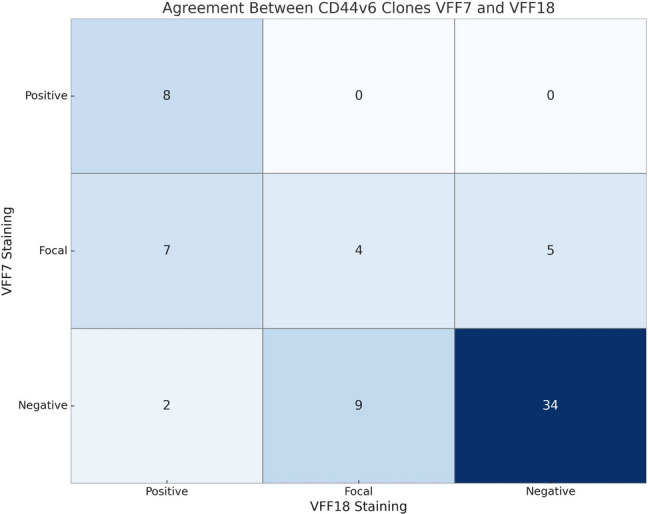
Agreement between CD44v6 immunoreactivity using VFF7 and VFF18 clones

The concordance between the two CD44v6 antibody clones (VFF-7, VFF-18) was evaluated by comparing immunohistochemical staining results in the validation cohort. There was moderate agreement between the two clones, with a Cohen’s weighted kappa coefficient of 0.66. Crude agreement between the two antibodies was 67%, indicating that two-thirds of the cases showed identical staining patterns. Staining results between antibody clones were concordant in 46 cases. However, some discrepancies were noted, most commonly when VFF-7 was negative and VFF-18 showed focal staining (9 cases), or when VFF-7 was focal and VFF-18 was positive (7 cases). Less frequent mismatches included focal VFF-7 with negative VFF-18 and negative VFF-7 with positive VFF-18 (Fig. 4).

### Messenger RNA expressional profiles in CD44v6 positive thyroid tumors

RNA sequencing data from the exploratory cohort (A) were used to perform gene expression analysis [16]. Out of 36 tumors, RNA-seq data were available for 32 cases, including 7 CD44v6-positive and 25 CD44v6-negative tumors. PCA based on global gene expression data was initially performed. This analysis did not reveal a clear separation between the two groups, likely due to both the limited number of CD44v6-positive cases and the histological similarity among the tumors (Supplementary Fig. 1A).

Furthermore, since our RNA-seq data did not specifically detect splicing variants such as the CD44v6 isoform, we examined the expression levels of the *CD44* standard form (*CD44*s). This analysis likewise did not reveal significant differences between CD44v6-positive and CD44v6-negative tumors at the mRNA level (Supplementary Fig. 1B). It should be emphasized, however, that these analyses are RNA-based and therefore do not directly reflect protein expression.

## Discussion

ATCs recurrently express CD44v6, and the potential clinical benefit of a radiolabeled antibody should not be underestimated. Several studies have demonstrated immunoreactivity of CD44v6 in ATC specimens and cell lines [30–33]. The primary objective of this study was to assess the prevalence of CD44v6 expression in non-ATC thyroid carcinomas that also present considerable clinical challenges. Accordingly, we examined a cohort of PTCs, OTCs and PDTCs, of which the two latter entities are particularly associated with substantial morbidity and mortality. Notably, up to half of the cases demonstrated CD44v6 positivity, either focally or diffusely. The presence of CD44v6 expression in non-ATCs underscores its potential as a therapeutic target, which would be particularly interesting in patients with limited radioiodine uptake, for whom treatment options remain scarce.

In this study, thyroid cancer samples were gathered retrospectively and divided into two cohorts based on whether they had radioiodine avidity data available (cohort A) or consisted of more clinically aggressive subtypes (cohort B). In the exploratory cohort (A), CD44v6 expression was observed in 30% of PTCs and 67% of PDTCs (two out of only three cases). The staining intensity and distribution varied, with some tumors showing focal areas of immunostaining while others exhibited diffuse positivity. This variability in CD44v6 expression probably reflects differences in tumor biology and underlying molecular mechanisms, with lower expression observed in tumors harboring *BRAF* mutations.

However, no significant differences in iodine avidity between CD44v6-expressing and non-expressing tumors were found, with cases overlapping between expression and avidity. *BRAF* mutations and radioiodine-refractoriness tend to be associated in PTC [34, 35], yet there is substantial overlap in quantitative avidity between *BRAF* mutated and wildtype lesions [36]. This may help explain the lack of a link in the current data and makes individual IHC analysis for CD44v6 expression imperative in radioiodine-refractive cases. In the validation cohort (B), similar patterns of CD44v6 expression were noted, with 50% of PDTCs and 38% of OTCs showing positivity.

Staining of the validation cohort with two different CD44v6 antibody clones enabled a comparative assessment of their staining patterns. Overall, concordance between the clones was observed in the majority of cases, however, some cases exhibited discordant results. Notably, VFF-18 stained a greater number of cases than VFF-7, often demonstrating low-level or borderline staining that VFF-7 either failed to detect or showed only focally. This apparent difference in staining patterns likely reflects intrinsic variations in epitope recognition and antibody affinity between the two clones.

The identification of CD44v6-positive tumors in non-ATCs highlights the potential for using CD44v6-targeted radionuclide therapy, such as [^1^⁷⁷Lu]Lu-DOTA-AKIR001 (NCT06639191), in patients with radioiodine-refractory disease. Given the limited treatment options for aggressive thyroid cancers, CD44v6-targeted therapies could provide a novel approach to improving patient outcomes. Since anti-CD44v6 CAR-NK therapy is being explored for various malignancies, our findings may also provide valuable insights for further investigations in this area [3, 37].

Despite the promising findings, the association between CD44v6 expression and clinical outcomes, including overall survival and disease-free survival, was not significant in our study. This indicates that while CD44v6 may serve as a useful biomarker for identifying candidates for targeted radiotherapy, its expression alone may not predict clinical outcome.

This study represents an exploratory mapping effort rather than a fully validated companion diagnostic. Several limitations should be acknowledged. First, the use of two CD44v6 antibody clones revealed a degree of discordance in staining sensitivity, which underscores the need for standardized assays in future clinical applications. Second, the *BRAF* mutation status was not available for all cases, limiting our ability to robustly correlate CD44v6 expression with molecular subtypes. Additionally, the absence of distinct clustering in RNA sequencing data between CD44v6-positive and -negative tumors may reflect underlying biological complexity, such as intratumoral heterogeneity or post-transcriptional regulation. Alternatively, it may simply indicate that CD44v6-expressing and non-expressing tumors are transcriptionally similar overall, with differences in CD44v6 expression arising from factors not captured at the global transcriptomic level, such as their divergent genetic backgrounds.

## Conclusion

In conclusion, our study shows CD44v6 expression in subsets of non-ATCs. CD44v6 expression was more frequent in *BRAF*-wildtype tumors, but did not appear strongly linked to iodine avidity. The identification of CD44v6-positive tumors may open new avenues for targeted radiotherapy for patients with aggressive and radioiodine-refractory thyroid cancer.

## Supplementary Information

Supplementary Material 1.

Supplementary Material 2.

## Data Availability

No datasets were generated or analysed during the current study.

## Abbreviations

ATC: Anaplastic thyroid carcinoma
PDTC: Poorly differentiated thyroid carcinoma
PTC: Papillary thyroid carcinoma
OTC: Oncocytic thyroid carcinoma
DHGTC: Differentiated high-grade thyroid carcinoma
IHC: Immunohistochemistry
FFPE: Formalin-fixed paraffin-embedded
RNA-seq: RNA-sequencing
TPM: Transcript per million
PCA: Principal component analysis
