# Supplementary material for: CD44v6 expression in non-anaplastic thyroid carcinoma: characterization of candidates for targeted therapy
Source: Thyroid Res. 2025 Oct 3;18:47. doi: 10.1186/s13044-025-00266-3 (PMC12492884; doi:10.1186/s13044-025-00266-3)
Supplement: Supplementary file 1 — Supplementary Material 1. [file 13044_2025_266_MOESM1_ESM.docx]

**Supplementary Material**

**Supplementary Figure 1.** Gene expression analysis comparing CD44v6-positive vs. negative cases. A. Principal component analysis (PCA) plot based on CD44v6-positive (n=7) and CD44v6-negative (n=25) tumors. B. Expression level of the standard variant of *CD44* reported as transcript per million (TPM) in CD44v6-positive vs. negative cases.
