# Supplementary figures and images for: CD44v6 expression in non-anaplastic thyroid carcinoma: characterization of candidates for targeted therapy
Source: Thyroid Res. 2025 Oct 3;18:47. doi: 10.1186/s13044-025-00266-3 (PMC12492884; doi:10.1186/s13044-025-00266-3)

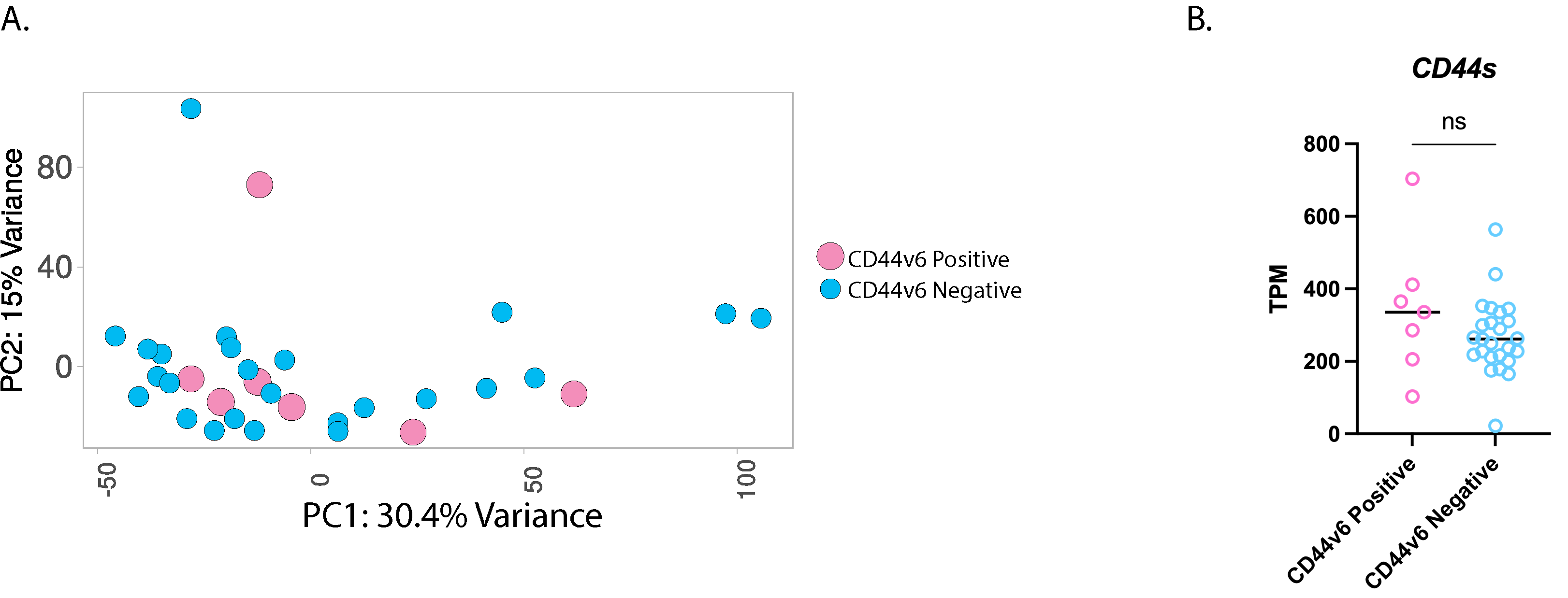

Supplement: Supplementary file 2 — Supplementary Material 2. [file 13044_2025_266_MOESM2_ESM.tif]
